# Abstracts/Sommaires/Zusammenfassungen

**DOI:** 10.1155/S1463924681000175

**Published:** 1981


### Abstracts Sommaires Zusammenfassungen

### Page 63

**Automated systems - the users' needs from a manufacturer's point of view**
T. M. Craig

The paper describes how a manufacturer in the healthcare field views users' requirements when developing automated systems for the clinical laboratory. The Du Pont Clinical Systems Division's automatic clinical analyser ('aca') illustrates the general principles manufacturers should consider when developing and marketing a new product.

**Systèmes automatiques — les besoins de l'utilisateur du point de vue du producteur**

Cette publication décrit comment un producteur dans le domaine de la santé publique voit les besoins de l'utilisateur en développant des systèmes automatiques pour le laboratoire clinique. L'analyseur automatique clinique ('aca') du département pour systèmes cliniques de Du Pont illustre les principes généraux, que le producteur devrait considérer en developpant et introduisant un nouveau produit.

**Automatisierte Systeme — die Erfordernisse des Benützers aus der Sicht des Herstellers**

Die Arbeit beschreibt wie ein Hersteller im Gebiet des Gesundheitswesens die Erfordernisse des Benützers sieht, wenn er automatisierte Systeme für das klinische Labor entwickelt. Der automatische klinische Analysator ('aca') der Du Pont Clinical Systems Divisions illustriert die allgemeinen Prinzipien, die ein Hersteller beim Entwickeln und Vermarkten eines neuen Produkts beachten sollte.

### Page 66

**Problems and possibilities of chemistry on dry reagent carriers**
J. Greyson

The paralleled approaches to layer chemistry are described and contrasted from both theoretical and practical aspects. The advantages and disadvantages of the two approaches are discussed. The effect these new technologies will have on the automated clinical chemistry world is outlined. Both technologies are seen as complementary approaches to clinical analysis.

**Problèmes et possibilités de la chimie sur supports de réactifs secs**

Deux approches parallèles à la chimie sur couches minces sont déscrits et comparés du point de vue théorique et pratique. Les avantages et désavantages des deux approches sont discutés. L'éffet de cette nouvelle technologie pour la chimie clinique automatisée est esquissé. Les deux technologies sont considérées comme approches complémentaires à l'analyse clinique.

**Probleme und Möglichkeiten der Chemie auf Trockenreagensträgern**

Die beiden parallelen Entwicklungsversuche zur Schichtchemie sind beschrieben und werden aus theoretischer und praktischer Sicht miteinander verglichen. Die Wirkung dieser neuen Technologien auf die automatisierte klinische Chemie wird skizziert. Beide Technologien werden als zueinander komplementäre Ansätze zur klinischen Analytik gesehen.

### Page 71

**Development of dry reagent chemistry for the clinical laboratory**
A. Zipp

The solid phase chemistry system developed by the Ames Company for clinical analysis is described and details given of the available chemistry systems. The system consists of a reflectance photometer, a Seralyzer, and chemistry for five analyses. The system was developed from the technology used in the urine strip products. The chemistry is carried on a solid phase reagent strip (a cellulose matrix) into which reagents are impregnated. Samples are presented to the strips, the reactions proceed and the measurements are carried out using the Seralyzer.

**Développement de la chimie sur supports de réactifs secs pour le laboratoire clinique**

Le système de chimie en phase solide développé par la Ames Company pour l'analyse clinique est décrit et les détails des systèmes chimiques disponibles sont donnés. Le système consiste en un photomètre à réflexion, un "Seralyzer" et la chimie pour cinq analyses. Le système a été développé à partir de la technologie utilisée pour indicateurs d'urine. La chimie a lieu sur une feuille à réactif en phase solide (matrice à cellulose) dans laquelle les réactifs ont été imprégnés. Les échantillons sont appliqués sur cette feuille, les réactions ont lieu et les mesures sont éffectuées en utilisant le "Seralyzer".

**Entwicklung der Trockenreagenschemie für das klinische Laboratorium**

Das Festphasenchemie-System, das durch die Ames Company für die klinische Chemie entwickelt wurde, wird beschrieben und Details zu den erhältlichen Chemie-Systemen werden angeführt. Das System besteht aus einem Reflexions-Photometer, einem Seralyzer, und Chemie für fünf Analysen. Das System wurde aus der bei den Urinstreifen-Produkten verwendeten Technologie heraus entwickelt. Die Chemie läuft auf einem Festphasenreagens-Streifen (eine Zellulose-Matrix) ab, die mit Reagentien getränkt werden. Die Probe wird auf den Streifen aufgezogen, die Reaktionen laufen ab, und die Messungen werden mit Hilfe eines Seralyzer durchgeführt.

### Page 76

**Microprocessor automation of a UV-visible monocrhomator**
M. W. Warren, et al

The design of a new controller for automation of a UV-visible spectrophotometer is presented that provides greatly improved performance compared to previous designs. The controller described can initialise, calibrate, and control both the wavelength and slitwidth. The control of an optional filter module is also discussed. The monochromator controller may be operated in a stand-alone mode from the keyboard/display or in a slave mode under the control of a master microprocessor through a serial I/O port. Most electromechanical errors can be automatically detected and reported. Although the design of a controller for a specific monochrometer is presented, the principles can be applied to other instruments.

**Automation d'un monochromateur UV-visible par microprocesseur**

Un nouveau système de contrôle pour l'automation d'un spectro-photomètre UV-visible qui fournit des performances nettement améliorées comparées aux anciens systèmes et présenté. Le systèm de contrôle décrit peut initialiser, calibrer et contrôler la longueur d'onde et la largeur de la fente. Le contrôle d'un module de filtre à option est également discuté. Le système de contrôle peut être opéré directement à partir d'un clavier et indicateur ou dans un mode esclave sous le contrôle d'un microprocesseur maître à travers un port sériel I/0. La plupart des erreurs électromécaniques sont détectées et rapportées automatiquement. Bien que le système de contrôle soit présenté pour un monochromateur spécifique, les principes peuvent être appliqués à d'autres instruments.

**Mikroprozessor-Automatisierung eines Monochromators im UV-sichtbaren Bereich**

Es wird eine neue Automatisierungssteuerung für ein Spektralphotometer im UV-sichtbaren Bereich beschrieben, das im Vergleich zu früheren Konstruktionen eine stark verbesserte Leistungsfähigkeit aufweist. Das beschriebene Steuergerät kann Wellenlänge und Spaltbreite initialisieren, kalibrieren und steuern. Die Steuerung eines zusätzlichen Filtermoduls wird ebenso diskutiert. Die Monochromatorsteuerung kann für sich allein von einer Tastatur mit Bildschirm betrieben werden oder über einen seriellen Ein-/Ausgang von einem übergeordneten Mikroprozessor ferngesteuert werden. Die meisten elektromechanischen Fehler können automatisch entdeckt und angezeigt werden. Obschon die Konstruktion eines Steuergerätes für einen spezifischen Monochromator vorgestellt wird, kann das Prinzip auch auf andere Geräte übertragen werden.

### Page 81

**Novel apparatus for the automation of solvent extraction**
J. G. Williams, et al

This paper presents a description of a novel apparatus which has successfully been developed and used for the automatic separation of an organic solvent from an aqueous phase. Separation is achieved by virtue of differences in density and surface tension between the liquids and is designed for minimum operator involvement necessitated by changes of solvent combinations. The method, unlike that used in many other solvent extraction systems, does not rely on the detection of phase boundaries between immiscible solvents.

**Nouvel instrument pour l'automation de l'extration par solvents**

Cette publication présente la description d'un nouvel instrument qui a été développé avec succès et utilisé pour la séparation automatique d'un solvent organique d'une phase aqueuse. La séparation est réalisée grace aux différences de densité et de tension de surface entre les liquides et l'instrument est concu pour un minimum de manipulations dues aux changements de combinaisons de solvents. La méthode, contrairement à celle utilisée dans beaucoup d'autres systèmes d'extraction par solvents, ne dépend pas de la détection des limites de phases entre deux solvents immiscibles.

**Ein neuartiges Gerät für die Automatisierung der flüssig-flüssig Extraktion**

Diese Arbeit beschreibt eine neuartige Apparatur, die erfolgreich für die automatische Trennung eines organischen Lösungsmittel von einer wässerigen Phase entwickelt und eingesetzt wurde. Die Trennung wird auf Grund von Unterschieden in Dichte und Oberflächenspannung der Flüssigkeiten erreicht und ist so konstruiert, dass nur minimale Bedienung beim Wechsel von Lösungsmittelkombinationen nötig ist. Die Methode, im Unterschied zu anderen flüssig-flüssig Extraktionssystemen, beruht nicht auf der Detektion der Phasengrenze zweier nicht mischbarer Lösungsmittel.

### Page 85

**Flexible data handling for routine quantitative analyses employing a gas chromatograph - mass spectrometer under computer control**
J. Vink. et al

A gas chromatograph - mass spectrometer operated under control of a Varian Spectro System 100 MS to perform quantitative analyses routinely has been extended. The standard software is modified with programs written in the simple language BASIC. Subroutines are developed to access data stored on disc after integration, to create and update a library of user-oriented programs, and to enable graphic display of mathematical functions. The subroutines are incorporated in BASIC programs which are stored on and loaded from magnetic tape. With the additional BASIC programs, the user defines interactively the datasystem criteria for data selection, calculation of calibration functions and calculation of final results of analyses.

**Traitement de donnees flexible pour analyses quantitatives de routine, utilisant un système GC-MS sous contrôle d'un ordinateur.**

Un système GC-MS contrôlé par un Varian Spectro System 100 MS, conçu pour analyses quantitatives de routine, a été élargi. Le software standard est modifié à l'aide de programmes écrits dans le language simple BASIC. Des sousprogrammes sont développés afin de donner accès aux données enregistrées sur disque après l'intégration, pour la création et la mise à jour d'un bibliothèque de programmes d'utilisateurs et afin de permettre la présentation graphique de fonctions mathématiques. Les sousprogrammes sont incorporés dan les programmes BASIC, enregistrés et disponibles sur bandes magnétiques. Les programmes supplémentaires BASIC permettent à l'utilisateur de définir de façon interactive les critères pour la sélection des données, le calcul des fonctions de calibrage et des résultats finals des analyses.

**Vielseitige Datenbearbeitung für quantitative Routineanalysen mit einem rechnergesteuerten GC-MS System**

Ein Gaschromatograph-Massenspektrometersystem, das von einem Varian Spectro System 100 MS gesteuert wird, um quantitative Routineanalysen durchzuführen, wurde erweitert. Die Standardsoftware wurde mit BASIC-Programmen modifiziert. Unterprogramme wurden entwickelt für den Datenzugriff auf dem Disk nach der Integration, für Erstellung und Unterhalt einer Bibliothek von Benützerprogrammen, und um eine graphische Darstellung von mathematischen Funktionen zu ermöglichen. Die Unterprogramme sind in BASIC-Programme eingebaut, welche auf Magnetband gespeichert sind und bei Bedar abgerufen werden können. Mit den zusätzlichen BASIC-Programmen kann der Benützer interaktiv die Datensystemkriterien festlegen für die Datenauswahl und für die Berechnung der Eichfunktionen und der Endresultate der Analysen.

### Page 88

**An evaluation of the Gemsaec 3E centrifugal analyser**
P. S. West, et al

The Gemsaec 3E is one of the latest centrifugal analysers. This report deals with its analytical performance in terms of precision, accuracy, calibration linearity and carryover for a number of tests based on different analytical principles viz. albumin by a dye binding method and by immunochemical precipitation, aspartate aminotransferase, creatine kinase, creatinine and triglyceride by kinetic methods, bilirubin by a bichromatic spectrophotometric method and total protein by conventional colorimetry. The precision for these tests was measured over a 20 day period and the data compared with other published data on centrifugal analysers and with published estimates of clinically allowable error. Accuracy was assessed by comparison of results with those obtained by established laboratory methods.

The analytical performance of the Gemsaec 3E was found to be acceptable by conventional standards. Comparison of patient results with those obtained by established laboratory methods showed a high degree of correlation with only small average differences insufficient to affect clinical judgement. The reasons for the differences are discussed and in some instances explained in terms of methodology.

**Evaluation de l'analyseur centrifuge Gemsaec 3E**

Le Gemsaec 3E est un des derniers analyseurs centrifuges. Ce rapport traite des performances analytiques en termes de précision, exactitude, linéarité au calibrage et contamination pour un nombre de tests basés sur différents principes analytiques, voir l'albumine par liaison de colorant et par précipitation immunochimique, l'aspartate aminotransferase, la créatine kinase, la créatinine et les triglycérides par des méthodes cinétiques, la bilirubine par une méthode spectrophotométrique bichromatique et la somme des protéines par colorimétrie conventionnelle. La précision pour ces tests était mesurée pendant une période de vingt jours et les données comparées avec d'autres données publiées pour analyseurs centrifuges, et comparées avec des estimations publiées sur l'erreur permise en clinique. L'exactitude a été déterminée par comparaison avec les résultats obtenus par des méthodes de laboratoire établies.

La performance analytique du Gemsaec 3E a été jugée acceptable selon les normes conventionnelles. La comparaison de résultats de patients avec ceux obtenus par des méthodes établies montre un haut degré de corrélation avec de petites différences moyennes insuffisantes pour affecter le jugement clinique. La raison pour les différences est discutée et pour certains cas expliquée en termes de méthodologie.

**Beurteilung des Gemsaec 3E Zentrifugal-Analysators**

Der Gemsaec 3E ist einer der neuesten Zentrifugal-Analysatoren. Dieser Bericht behandelt die analytische Leistung, ausgedrückt in Zahlen zur Präzision, Genauigkeit, Kalibrierungs-Linearität und Verschleppung für eine Anzahl von Analysen. Diese basieren auf verschiedenen analytischen Principien wie z.B. Albumin-Bestimmung mit einer Farbstoffbindungs-Methode und durch immunochemischen Niederschlag, Aspartat-Aminotransferase, Kreatin-Kinase, Kreatinin und Triglyzerid mit kinetischen Methoden, Bilirubin mit einer bichromatischen, spektrophotometrischen Methode und Gesamt-Protein durch konventionelle Kolorimetrie. Die Präzision für diese Analysen wurde über eine Periode von 20 Tagen gemessen und die Daten mit anderen veröffentlichten Daten zu Zentrifugal-Analysatoren und mit veröffentlichten Schätzungen von klinisch zulässigen Fehlern verglichen. Die Genauigkeit wurde durch einen Vergleich der Resultate mit solchen, die mit etablierten Labormethoden erhalten werden, beurteilt.
Die analytische Leistungsfähigkeit des Gemsaec 3E wurde nach konventionellen Masstäben als akzeptabel befunden. Ein Vergleich von Patientendaten mit solchen, die mit etablierten Labormethoden erhalten werden, zeigen einen hohen Korrelationsgrad mit nur kleinen durchschnittlichen Abweichungen, die ein klinisches Urteil nicht beeinflussen. Die Ursachen für die Abweichungen sind diskutiert und in einigen Fällen methodologisch erklärt.

### Page 92

**Computer evaluation of the EMIT assays Carbamazepine, Ethosuximide, Phenobarbital, Phenytoin, Quinidine and Theophylline on the Gemsaec Centrifugal Fast Analyser.**

B. Kinberger and Bengt-Ake Johansson

An automated procedure is described for the EMIT drug assays on the Gemsaec centrifugal fast analyser. The transfer disc is prepared by totally automatic pipetting. All the calculations are performed with the Gemsaec computer (PDP 8/e) using a specially designed FOCAL program. Calibration factors, as computed from standard curves, were stored on magnetic tape. Economical runs were then performed using only two non-zero calibrations per disc. The stored standard curve was checked and recalibrated if needed. Twelve positions were left free on each disc for the evaluation of unknown samples. The sample throughput is increased by about 30% as compared to a procedure using a "full set" of calibrations in each run.

**Evaluation par ordinateur si EMIT permet d'analyser la Carbamacépine, l'Ethosuximide, le Phénobarbital, la Phénytoin, la Quinidine et la Théophylline sur l'analyseur centrifuge rapide Gemsaec**

Une procédure automatisée est décrite pour analyses EMIT de produits pharmaceutiques par l'analyseur centrifuge rapide Gemsaec. Le disque de transfert est préparé par pipettage automatique. Toutes les calculations sont exécutées par l'ordinateur Gemsaec (PDP 8/e), à l'aide d'un programme spécial FOCAL. Les facteurs de calibration, calculés à partir de courbes standard, sont fixés sur bande magnétique. Des mesures économiques sont obtenues en employant seulement deux calibrages non-zéro par disque. La courbe standard enregistrée est contrôlée et recalibrée si nécessaire. Douze positions sont restées vacantes sur chaque disque pour l'évaluation d'échantillons inconnus. Le débit d'échantillons peut de cette façon être augmenté d'environs 30% par rapport à une procédure utilisant le jeu complet de calibrage.

**Computer-Bewertung der EMIT-Analysen von Karbamazepin, Ethosuximid, Phenobarbital, Phenytoin, Chinidin und Theophyllin auf dem Gemsaec Zentrifugal-Schnellanalysator**

Ein automatisiertes Vorgehen für EMIT von Pharmazeutika auf dem Gemsaec Zentrifugal-Schnellanalysator ist beschrieben. Der Transferteller wird mit vollständig automatisiertem Pipettieren vorbereitet. Alle Berechnungen werden auf dem Gemsaec-Rechner (PDP 8/e) mit Hilfe eines speziellen FOCAL-Programms durchgeführt. Eichfaktoren, wie sie aus Standardkurven berechnet werden können, werden auf Magnetband abgespeichert. Einsparende Messreihen wurden aufgenommen, bei denen nur zwei Eichungen pro Teller benützt wurden. Die abgespeicherte Standardkurve wurde überprüft und falls notwendig wurde wieder geeicht. Zwölf Positionen pro Probenteller blieben verfügbar für die Analyse unbekannter Proben. Der Probendurchsatz ist um ca. 30% erhöht im Vergleich zu einem Vorgehen, das pro Messreihe einen "vollen Satz" von Eichungen benützt.

### Page 97

**Further electrode evaluation of the Nova 2 ionised calcium instrument**

J. A. Fyffe, et al

Following the previous studies where differences in the calcium ion-selective electrodes of the Nova 2 were reported, six electrodes have now been compared. Differences are shown in practical reponse time to serum and in results of replicate assay of serum samples by different electrodes. The six electrodes are identical in calibration curve slope, detection limit, potentiometric selectivity coefficients and response time to aqueous media.

**Evaluation d'autres électrodes pour l'instrument Nova 2 pour calcium ionisé**

A la suite d'études antérieures nous avons rapporté des différences entre les électrodes à ions spécifiques du Nova 2, six électrodes ont maintenant été comparées. Les différences dans le temps de réponse pratique pour le sérum et les résultats d'essais répétés sont démontrées pour des échantillons de sérum en utilisant différentes électrodes. Les six électrodes étaient identiques en ce qui concerne la pente de la courbe de calibration, la limite de détection, les coëfficients de sélectivité potentiométrique et le temps de réponse en milieu aqueux.

**Weitere Elektroden-Evaluation des Instruments NOVA 2 für "Ionisiertes Kalzium"**

Als Folge zu vorgängigen Studien die über Unterschiede der ionenselektiven Kalzium-Elektroden mit dem Nova 2 berichteten, wurden jetzt sechs Elektroden verglichen. Unterschiede werden aufgedeckt bezüglich der Reaktionszeit auf Serum und bezüglich der Resultate identischer Analysen von Serumproben mit verschiedenen Elektroden. Die sechs Elektroden sind identisch bezüglich Steigung der Eichkurve, Detektionsgrenze, potentionmetrische Selektivitätskoeffizienten und Reaktionszeit in wässrigen Medien.

